# Highly active engineered IgG3 antibodies against SARS-CoV-2

**DOI:** 10.1073/pnas.2107249118

**Published:** 2021-10-01

**Authors:** Somanath Kallolimath, Lin Sun, Roman Palt, Karin Stiasny, Patrick Mayrhofer, Clemens Gruber, Benjamin Kogelmann, Qiang Chen, Herta Steinkellner

**Affiliations:** ^a^Department of Applied Genetics and Cell Biology, University of Natural Resources and Life Sciences, 1180 Vienna, Austria;; ^b^Center for Virology, Medical University of Vienna, 1090 Vienna, Austria;; ^c^Department of Biotechnology, University of Natural Resources and Life Sciences, 1180 Vienna, Austria;; ^d^Core Facility Mass Spectrometry, University of Natural Resources and Life Sciences, 1180 Vienna, Austria;; ^e^The Biodesign Institute and School of Life Sciences, Arizona State University, Tempe, AZ 85281

**Keywords:** engineered IgG3, plant-based expression, antibodies, SARS-CoV-2, virus neutralization

## Abstract

Monoclonal antibodies (mAbs) that efficiently neutralize SARS-CoV-2 have been developed at an unprecedented speed. Notwithstanding, there is a vague understanding of the various Ab functions induced beyond antigen binding by the heavy-chain constant domain. To explore the diverse roles of Abs in SARS-CoV-2 immunity, we expressed a SARS-CoV-2 spike protein (SP) binding mAb (H4) in the four IgG subclasses present in human serum (IgG1-4) using glyco-engineered *Nicotiana benthamiana* plants. All four subclasses, carrying the identical antigen-binding site, were fully assembled in planta and exhibited a largely homogeneous xylose- and fucose-free glycosylation profile. The Ab variants ligated to the SP with an up to fivefold increased binding activity of IgG3. Furthermore, all H4 subtypes were able to neutralize SARS-CoV-2. However, H4-IgG3 exhibited an up to 50-fold superior neutralization potency compared with the other subclasses. Our data point to a strong protective effect of IgG3 Abs in SARS-CoV-2 infection and suggest that superior neutralization might be a consequence of cross-linking the SP on the viral surface. This should be considered in therapy and vaccine development. In addition, we underscore the versatile use of plants for the rapid expression of complex proteins in emergency cases.

Pre- and postexposure immunotherapies with neutralizing antibodies (nAbs) are presently being explored for the prevention and treatment of COVID-19. This has, at an unprecedented speed, led to the isolation of mAbs that efficiently neutralize SARS-CoV-2 (e.g., H4) (1). These mAbs, mainly directed against spike protein (SP) on the virion`s surface, are predominantly of the IgG1 subtype. However, a strong prevalence of other subclasses (like IgG3 and IgG4) was observed in the sera of COVID-19 patients (2), indicating a protective effect during the viral infection. While the role of IgG1 is intensively investigated, data on the functional impact of the other IgG subclasses, discriminated mainly by diverse Fc domains, are rare. Notably, the first SARS-CoV-2 studies on distinct IgG-Fc functional profiling point to enhancement of disease (3), in line with data from SARS-CoV experiments (4). These data underscore the need for a better understanding of the molecular properties of SARS-CoV-2 Ab variants prior to application in therapy or prophylaxis.

To further explore the role of Ab domains beyond antigen-binding in SARS-CoV-2 immunity, we focused on the expression and functional analyses of a neutralizing mAb with identical variable domains but different constant heavy chains (HCs), representing all four IgG subclasses present in human serum (IgG1-4).

## Results

### Production of Recombinant Monoclonal H4-IgG Subclasses.

**Fig. 1.**
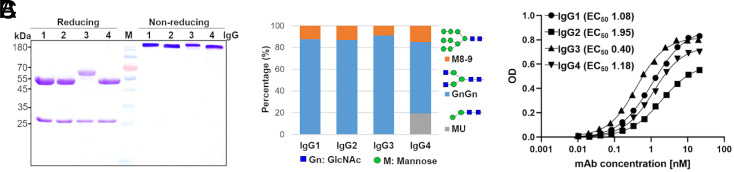
Biochemical characterization and antigen-binding activities of recombinant H4-IgG1-4 produced in ΔXTFT. (*A*) Purified H4-IgG1-4 separated by reducing and nonreducing SDS/PAGE (Coomassie brilliant blue stained), 4 µg protein was loaded at each lane. (*B*) LC-ESI-MS/MS–derived glycosylation profiles of purified H4-IgG1-4. Bars represent the relative abundance (%) of glycoforms present at the conserved Fc glycosite. (*C*) ELISA-binding activity EC_50_ values in nanomoles of purified H4-IgG1-4 to SP using antibodies against κ-LC for detection.

H4 SARS-CoV-2 neutralizing monoclonal IgG1 antibody that binds to an epitope at the receptor-binding domain of the SP (1) served as a template in this study. Four plant expression constructs with identical variable regions but different HC domains, representing human subclasses IgG1-4, were generated (*H4-IgGHC1-4*) (*SI Appendix*). The IgG3 isotype refers to allotype G3m5, containing a 62-amino-acid hinge domain. For light-chain expression, a single construct carrying the variable region of H4 and κ-light chain constant (κ-LC) domain was used for all subclasses (*H4-IgGLC)*. Respective vectors of *H4-IgGHC1-4* and *H4-IgGLC* were coexpressed in *Nicotiana benthamiana* glycosylation mutant ΔXTFT by agroinfiltration (5, 6) and purified at 4 d postinfiltration by protein A–based affinity and subsequent size-exclusion chromatography. SDS/PAGE analysis of purified mAbs revealed the correct expression, assembly, and high purity of all four subclasses (Fig. 1*A*).

### Glycosylation Status of Plant-Derived H4-IgG Subclasses.

It is well known that the conserved Fc glycosylation at position N297 plays a vital role in Fcγ receptor (FcγR) interactions and effector function. In line, recent results demonstrate glycosylation-dependent functional effects during COVID-19 (7). Thus, we aimed at the production of mAbs with a targeted glycosylation profile. Liquid chromatography–electrospray ionization–tandem mass spectrometry (LC-ESI-MS/MS) (*SI Appendix*) of H4-IgG1-4 revealed a largely homogeneous Fc glycosylation profile, with ∼90% GnGn structures (i.e., core xylose and fucose-free *N*-glycans) (Fig. 1*B*).

### Antigen-Binding and Neutralization Potency of H4-IgG Subclasses.

Antigen-binding properties of H4-IgG1-4 were determined by ELISA using Chinese hamster ovary–produced SARS-CoV-2 SP. The following binding activities, expressed by EC_50_ values, were obtained: IgG2 < IgG1 = IgG4 < IgG3 (EC_50_ 1.95, 1.08, 1.18, and 0.4 nM, respectively) with an up to fivefold increased binding of IgG3 compared to the lowest binder, IgG2 (Fig. 1*C*).

To further determine functional activities of Ab subclasses, Vero cell–based SARS-CoV-2 neutralization (NT) assays were performed, as described previously (8) (*SI Appendix*). IgG1, IgG2, and IgG4 exhibited similar NT potencies (NT_100_ 295.88, 296.49, and 222.36 nM, respectively) (Table 1). However, IgG3 displayed an up to 50-fold increased activity (NT_100_ 5.91 nM) (Table 1) in comparison with the other subclasses. Our results point to an important role of HC domains that are not directly involved in antigen binding.

**Table 1.** SARS-CoV-2 NT activities (NT_100_ values) of H4-IgG subclasses

Ab subtype	NT_100_ (nM)
H4-IgG1	295.88
H4-IgG2	296.49
H4-IgG3	5.91
H4-IgG4	222.36

## Discussion

While major differences in IgG subtype prevalence in human sera during and after viral infections compared to healthy individuals are frequently observed, the consequences thereof are not well understood. Here we performed a side-by-side comparison of a SARS-CoV-2 neutralizing mAb with identical antigen-binding domains but four different HCs. Our results demonstrate substantially increased NT activities of IgG3 compared to the other three subclasses, despite relative modest variations in antigen binding.

Major differences of IgG3 compared to the other IgG subclasses are the long *O*-glycosylated hinge region and single point mutations in the Fc domain that enable, for example, high-affinity interaction with activating FcγRs. Such characteristics make IgG3 a uniquely potent immunoglobulin, with the potential for triggering effector functions and enhanced viral NT (9). Since we used Vero cells that do not carry FcγR in our studies, superior SARS-CoV-2 NT induced by Fc-mediated effector activities can be excluded. More likely, our data suggest that the unusual hinge region of IgG3 is a key factor that mediates altered activities. Indeed, previous studies, particularly in HIV settings, report a significant impact of the hinge region on the Ab’s functional activities (10). It has been suggested that augmented Ab-based NT potency toward HIV is driven by “intratrimeric cross-linking” of the two Fab arms, thus increasing the overall avidity of the spike–Ab interaction (11, 12). Our data are in line with such studies and indicate that enhanced SARS-CoV-2 NT is a consequence of cross-linking SP on the viral surface, induced by the unique structural features of IgG3. Also in accordance with HIV data (11), we hypothesize that cross-linking lowers the concentration of Abs required for NT and low spike densities facilitate Ab evasion. Interestingly, the SP copy number per SARS-CoV-2 virion is comparable with HIV, but 5 to 10 times less than that of other enveloped viruses, such as the influenza virus (13, 14). Notably, epitope-specific induction of neutralizing IgG3 Abs has been reported (e.g., HIV, Chikungunya virus) (9). This is an additional factor that cannot be excluded in our studies.

In addition, we show the expression of Ab variants with a single dominant *N*-glycan species (i.e., GnGn or G0). This glycan form, known to confer proinflammatory activities on Abs, is detected only in minor amounts in human-derived Abs. Here, we do not expect functional alterations due to this modification; however, fucose-free SARS-CoV-2 Abs may exhibit increased in vivo activities. This needs to be proven. In addition, the conserved core GnGn structures (5) simplify further engineering to elucidate so far potentially unknown glycosylation-dependent activities of SARS-CoV-2 antibodies, as suggested recently (7). Collectively, our results deliver insights into SARS-CoV-2 immunology and should perchance be considered for therapy or passive SARS-CoV-2 protection.

## Materials and Methods

In planta expression of IgG subtypes was accomplished by agroinfiltration of respective DNA constructs. Subsequently, affinity- and size exclusion chromatography–purified mAbs were subjected to ELISA antigen-binding assays and NT activity was measured by a Vero cell–based SARS-CoV-2 infection assay, as described recently (8). Materials and methods are detailed in *SI Appendix*, *Supplementary Text Materials and Methods*.

## Data Availability

All study data are included in the article and *SI Appendix*.
